# The Efficacy of Telemental Health Interventions for Mood Disorders Pre-COVID-19: A Narrative Review

**DOI:** 10.1007/s11414-024-09884-5

**Published:** 2024-05-02

**Authors:** Alyssa M. Edwards, Jordan C. Petitt, Martha Sajatovic, Sanjana Kumar, Jennifer B. Levin

**Affiliations:** 1 Case Western Reserve University School of Medicine, Cleveland, OH USA; 2 Department of Psychiatry, University Hospitals Cleveland Medical Center, W.O. Walker Bldg, 7th Floor, 10524 Euclid Ave, Cleveland, OH 44106 USA

## Abstract

The onset of the COVID-19 pandemic saw a significant surge in the utilization of telemental health (TMH) services. This narrative review aimed to investigate the efficacy of TMH for serious mood disorders prior to the COVID-19 pandemic. A search across databases was conducted for randomized controlled trials focusing on TMH interventions for mood disorders, encompassing major depressive disorder (MDD) and bipolar disorder (BD). Study and patient characteristics, interventions, and outcomes were extracted. From a pool of 2611 papers initially identified, 17 met the inclusion criteria: 14 focused on MDD, while 4 addressed BD. Among these, 6 papers directly compared TMH interventions to in-person of same treatment, revealing improved access to care and higher rates of appointment follow-up with TMH. Additionally, 6 papers comparing TMH to treatment as usual demonstrated improvements in mood outcomes. Conversely, 3 papers comparing different TMH interventions found no discernible differences in outcomes. Notably, 3 studies evaluated TMH as an adjunct to usual care, all reporting enhancements in depression outcomes. Overall, preliminary evidence suggests that prior to COVID-19, TMH interventions for serious mood disorders facilitated improved access to care and follow-up, with comparable clinical outcomes to traditional in-person interventions. The discussion addresses limitations and provides recommendations for future research in this domain.

**Supplementary Information:**

The online version contains supplementary material available at 10.1007/s11414-024-09884-5.

## Introduction

The US National Institute of Mental Health (NIMH) defines telemental health (TMH) as “the use of telecommunications or videoconferencing technology to provide mental health services.”^1^ TMH can provide a range of services including psychiatric evaluations, therapy, patient education, and medication management through a live and interactive technological interface. Given the limited workforce in psychiatry, even prior to COVID-19, there has been a higher demand, compared to the available supply of mental health providers and resources.^2^ TMH offers efficient use of scarce resources. Since its inception, TMH has improved access to mental health care, allowed healthcare providers to treat more patients in distant locations, and has contributed to patient satisfaction.^3^

The use of videoconferencing in the field of psychiatry began in 1959 when the Nebraska Psychiatric Institute utilized videoconferencing to provide group therapy, long-term therapy, consultation-liaison psychiatry, and medical student training at the Nebraska State Hospital in Norfolk.^4^ TMH grew in popularity throughout the 1970s and 1980s and eventually spread across the world in the 1990s.^4^ As it became more commonly used, researchers began to evaluate TMH’s ability to facilitate access to care, overcome geographical obstacles, and how it compared to in-person care. By the 2000s, it became clear that TMH can be effective, but is distinctly different from in-person care.^4^ In February 2018, the American Psychiatric Association (APA) updated its public recommendations, stating “Telemedicine in psychiatry, using videoconferencing, is a validated and effective practice of medicine that increases access to care.”^3^

During the COVID-19 public health emergency (PHE), there was a dramatic increase in the use of TMH services.^5^ In June 2020, near the beginning of the COVID-19 pandemic, the APA conducted a survey that included a question regarding the use of TMH prior to the COVID PHE. Of the 600 psychiatrist respondents, 64% reported not seeing any of their patient caseload via TMH health prior to the PHE.^5^ In contrast, when the survey was repeated in January 2021, 81% of respondents reported that they were seeing the vast majority of their patients (between 75 and 100%) via TMH, with 39% reporting transitioning back to seeing at least some patients in-person.^5^

In addition to psychiatrists, diverse mental health professionals including psychologists, counselors, social workers, and psychiatric nurses have increased their use of TMH service delivery.^2^ Considering the dramatic shift towards utilizing TMH, it was of interest to investigate the body of research on the efficacy of TMH interventions for the treatment of serious mood disorders that existed prior to the COVID-19 pandemic. While there is some evidence of the efficacy of TMH for the treatment of PTSD, anxiety, and ADHD, the results cannot necessarily be generalized to individuals with serious mood disorders.^6–8^ Care for individuals with serious mood disorders often includes evaluation of risk to self and can involve multiple treatment modalities such as medication and behavioral therapies. A recent meta-analysis found a limited number of randomized controlled trials in TMH research focused on mood disorder treatments.^9^

The objective of this narrative review was to assess the efficacy of TMH for the treatment of individuals with serious mood disorders, including major depressive disorder (MDD) and bipolar disorder (BD), a subset of serious mental illness, prior to the onset of the COVID-19 pandemic.

## Methods

### Study Selection

Pubmed, PsycInfo, Cochrane Collaboration, Web of Science, and CINAHL databases were searched for original research reports in the English language up to April 2020, prior to the COVID-19 pandemic, on the efficacy of TMH interventions for patients with serious mood disorders. Each source was searched and consulted on March 10, 2021. The following query terms were used in for the Pubmed database search: (telemedicine[mesh] OR telemed*[tiab] OR telehealth*[tiab] OR telepsych*[tiab] OR teleconferenc*[tiab] OR teleconsult*[tiab] OR remote deliver*[tiab] OR remote consult*[tiab] OR virtual deliver*[tiab] OR virtual consult*[tiab]) AND (“serious mental illness*”[tiab] OR “severe mental illness*”[tiab] OR “depressive disorder, major”[mesh] OR major depression[tiab] OR major depressive[tiab] OR “bipolar and related disorders”[mesh] OR bipolar[tiab]) AND english[lang]). The complete search strategy for each database is included in Appendix 1. The search included combinations of the following keywords: telemedicine, telehealth, telepsychiatry, remote delivery, virtual delivery, serious mental illness, severe mental illness, major depression, and bipolar and related disorders. Additionally, a review of the bibliographies of the final pool of articles was conducted to identify any additional relevant articles.

Serious mood disorders were defined as bipolar disorder (BD) or major depressive disorder (MDD), with diagnosis determined based on patient self-report, clinical evaluation, standardized diagnostic interview, or medical record review. Papers that solely classified cases based on symptom reports or depression severity scale usage (e.g., PHQ-9) were excluded. Only randomized controlled trials (RCT) conducted pre-COVID with at least one patient-level outcome were included. Papers including other psychiatric or medical conditions were eligible if subgroup analyses of individuals with a serious mood disorder diagnosis were reported. Interventions where the delivery was primarily or entirely remote by a clinician (e.g., doctor, nurse, social worker, counselor) using interactive real-time technology, or via telephone, were included.

Exclusion criteria covered four categories: diagnosis, type of TMH intervention, patient outcomes, and study design. Interventions primarily focusing on substance use/abuse or other conditions not classified as serious mood disorders, such as post-traumatic stress disorder or premenstrual dysmorphic disorder, were excluded. Papers evaluating a mood disorder but lacking separate analyses of BD or MDD samples were also excluded, as were primarily automated therapies or apps.

All abstracts underwent relevance screening by three authors (AE, JP, JL), excluding systematic reviews, retrospective studies, and case reports. To ensure that the inclusion and exclusion criteria were being applied in the same way, three of the authors performed a dry run of abstract review. The first 10 abstracts from PubMed were compiled into an Excel spreadsheet, and the authors independently determined whether the papers met inclusion/exclusion criteria. The three authors then compared decisions and reached consensus on any discrepancies before applying uniform criteria to the remaining papers.

Papers were categorized into four different comparison types: (1) direct comparison of TMH vs same treatment delivered in-person, (2) TMH + In-person vs In-person alone (TMH as add on to usual care), (3) TMH intervention versus in-person standard care control, and (4) TMH vs another telehealth control (such as telephone-delivered calls recalling neutral events). 

### Analytical Strategies

A data extraction checklist was developed to code study characteristics including author, year, study design, sample description, control group, intervention, outcome measurement, and results. To rate the methodological quality of papers, a modified version of the Methodological Quality Rating Scale (MQRS) was employed.^10^ This rating scale assesses the methodological quality of papers across 12 different dimensions (for example, study design and follow-up length). Cumulative MQRS scores for each study range from one (poor quality) to 16 (high quality). A score of 14 or higher is considered well-designed, 7–13 is moderate, and a score of 1–6 is considered low quality.

## Results

### Study Selection

**Figure 1:**
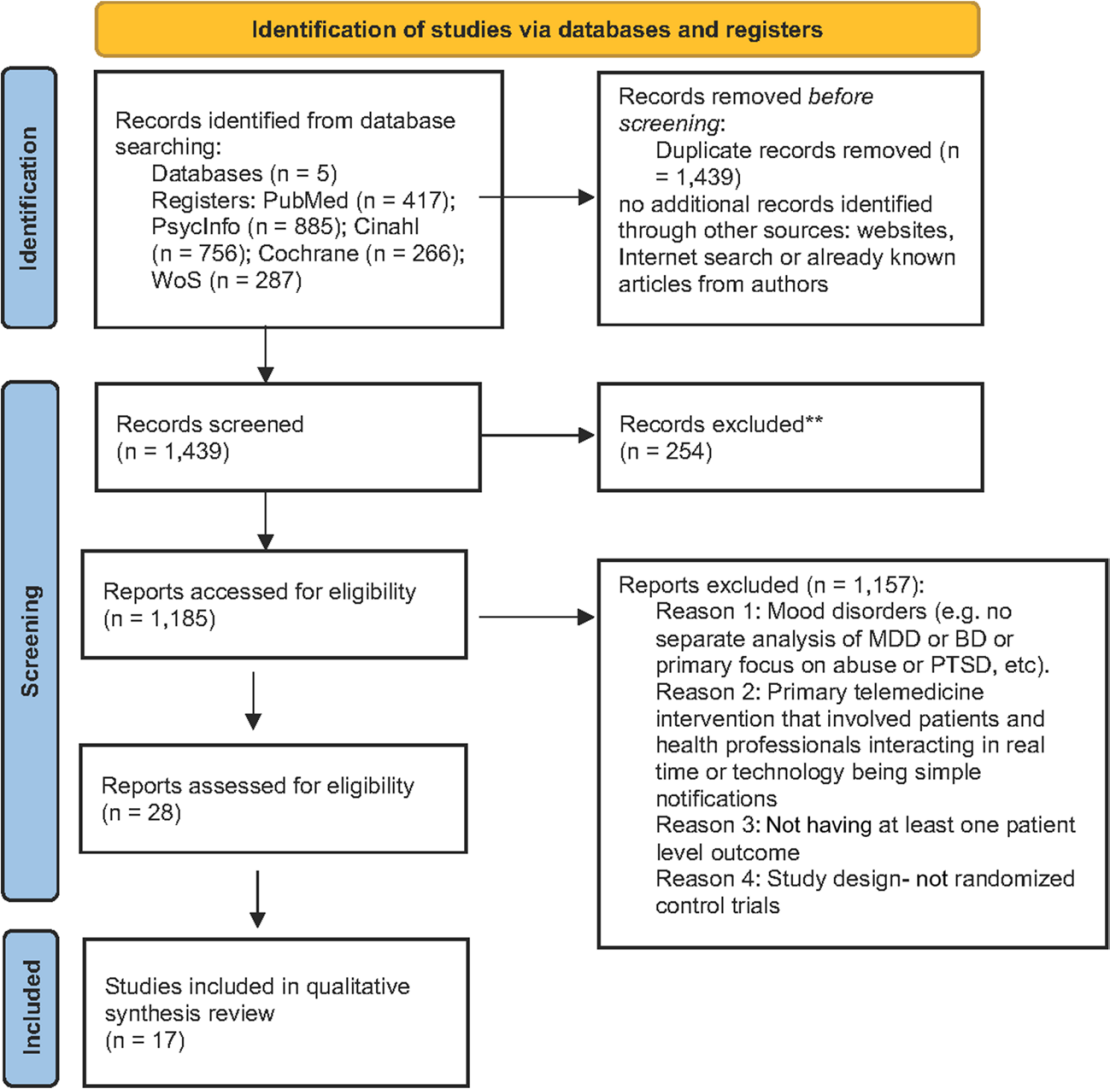
Flow diagram of the study selection process. Adapted PRISMA flow diagram (2020) (http://www.prisma-statement.org/)

The literature search returned a total of 2611 papers. After removal of 1172 duplicates, a total of 1439 publications were screened. Following abstract and title screening as well as scanning the references of selected papers, 28 papers underwent full-text screening and data extraction, and 17 met inclusion criteria and were included in the final narrative review (Fig. 1). Of the 17 included papers, there were only 16 original studies as two papers were based upon the same study.^11,12^

### Study Design

The study design of all the included studies was RCTs, ranging in duration from 4 weeks to 18 months.^12,13^ In all studies, the RCTs had two-arm comparisons: (1) direct comparison of TMH vs same treatment delivered in-person, (2) TMH + In-person vs In-person alone (TMH as add on to usual care), (3) TMH intervention versus in-person standard care control, and (4) TMH vs another telehealth control (such as telephone-delivered calls recalling neutral events). Six of the papers compared the same treatment delivered via TMH versus in-person interventions (35.3%).^11,12, 14–17^ Three papers compared an add-on TMH treatment to usual care vs usual care (17.6%).^18–20^ Five of the papers compared specific telemental health treatment to in-person control (29.4%).^21–25^ Three compared one telemental health treatment to another telehealth control, two of which were telephone-based and one video-telephone hybrid (17.6%).^13,26, 27^  

Interventions included communication between a provider and a patient via electronic means such as telephone, videoconference, or other digital media platforms. All 17 papers used digital communication either through telephone (58.9%) or webcam/videoconferencing (41.2%). The control for the 3 studies that compared 2 TMH interventions included telephone calls recalling neutral events, adherence reminder telephone calls, or telephone-based disease management modules.^13,26, 27^

Descriptions of the interventions, controls, outcomes, and results are displayed in Table 2. Schulze described usual care as occasional in-person physician visits to evaluate illness compared to TMH as add-on to usual care.^18^ Chong used a webcam that consisted of monthly TMH visits provided by a psychiatrist using an online virtual meeting program.^15^ Both Lam and Schulze included components consisting of adherence-reminder telephone calls from trained nursing staff as part of their TMH intervention delivered via phone but had different comparison groups.^26,18^ Between the video-based and telephone-based subgroups, no outcome differences were seen (Tables 1 and 2).^26,18^

**Table 1:** Study characteristics—demographics, setting, and duration of randomized controlled trial testing telemental health interventions in patients with serious mood disorders

Study (first author, publication year)	Mean age (years)	Sex (% female)	Sample size (*n*)	Population	Location	Study setting	Study design and duration	Methodological Quality Ratings Scale (MQRS)
Dennis (2020)	-	100%	241	Postpartum women	Canada (rural and urban)	Public health departments from 36 health regions in 6 provinces in Canada	36-week prospective trial of standard postpartum care vs telephone interpersonal psychotherapy (IPT) + standard care	9
Celano (2020)	45.4	68%	25	Inpatients with bipolar depression	United States (urban)	Inpatient psychiatric unit	4-week prospective trial of telephone-delivered neutral phone calls vs telephone-delivered positive psychology (PP)	12
Chong (2011)	43.0	88%	167	Low-income Hispanic patients with MDD	United States-Mexico Border (rural)	Community health center (CHC)	6-month prospective trial of webcam-delivered telepsychiatry (WEB) vs treatment as usual (TAU)	10
D’Souza (2002)	47.9	43.1%	51	Patients with bipolar affective disorder	Australia (rural)	Inpatients admitted to a tertiary psychiatric center	12-month prospective trial of videoconference discharge planning and sessions of psycho-educational program vs conventional discharge summaries and no program	7
Egede (2015)*	63.9	2.5%	241	Veterans ≥ 58 years with MDD	United States (Southeast)	Ralph H Johnson Veterans Affairs Medical Center and 4 associated community outpatient-based clinics	12-month prospective trial of telemedicine MDD treatment vs in-person treatment	13
Egede (2016)*								11
Fann (2015)	45.8	37%	100	Patients with MDD within 10 years of complicated mild to severe TBI	United States	Clinical and community settings	16-week telephone-delivered cognitive behavioral therapy (T-CBT) vs in-person	9
Lam (2013)	43.3	54.5%	99	Employed patients with MDD	Canada (Vancouver, Calgary, Toronto)	Clinical referrals and advertising	12-week prospective trial of telephone-administered cognitive-behavioral therapy (T-CBT) vs adherence-reminder telephone calls	10
Mohr (2011)	55.9	9.4%	85	Veterans with MDD	United States (rural California and rural Illinois)	Community-based outpatient clinic	20-week prospective trial of T-CBT vs TAU	9
Schulze (2019)	42.9	41.7%	120	Outpatients with schizophrenia or bipolar disorder	Germany	120 patients from 3 inpatient or day care unit psychiatric hospitals	6-month prospective trial of medication adherence telephone calls vs TAU	9
Yeung (2016)	50	63%	190	Chinese Americans with MDD	United States (South Cove, MA)	South Cove Community Health Center—Primary Care Setting	6-month prospective trial of telepsychiatry vs TAU	10
Luxton (2016)	-	18.2%	121	U.S. military service members and veterans with depression	United States (WA and Portland, OR)	Joint Base Lewis-McChord (Washington State) and VA Health Care System (Portland, Oregon)—Large Regional Military Facilities	3-month prospective trial of teleconference behavioral activation treatment for depression (BATD) vs in-person BATD	9
Heckman (2017)	51.9	37%	132	HIV-infected rural persons with MDD, partially remitted MDD, or dysthymic disorder	United States (rural)	Brochures from AIDS service organizations in 28 states	9-week prospective trial of telephone IPT vs standard care	7
Dobkin (2020)	65.2	51.4%	72	Patients with Parkinson’s disease (PD) and depressive disorder	United States	Tertiary care Departments of Neurology and Psychiatry, PD support groups, Clinical Trial Match Tool	6-month prospective trial of T-CBT vs TAU	9
Kalapatapu (2014)	43.7	87.4%	103	Patients with depression and problematic alcohol use	United States	Primary care patients	6-month prospective trial of T-CBT vs TAU	9
Hilty (2007)	46	80%	94	Patients with MDD	United States (rural CA)	Eight rural primary care sites with 17 clinics	12-month prospective trial of telephone intensive disease management module (IDMM) and tele-video psychiatric consultation vs TAU	13
Hulsbosch (2017)	46	48%	93	Patients with depression and bipolar disorder, schizophrenia and other psychotic disorders, or other	Netherlands (Northwestern)	Two community mental health teams	18-month prospective trial of videoconferencing vs TAU	10

^*^Egede (2015) and Egede (2016) papers report on different outcomes from the same study

Abbreviations: *BATD* behavioral activation treatment for depression; *CHC* community health center; *IDMM* intensive disease management module; *IPT* interpersonal psychotherapy; *NA* not available; *MDD* major depressive disorder; *PP* positive psychology; *TAU* treatment as usual; *T-CBT* telephone-administered cognitive behavioral therapy; *TBI* traumatic brain injury; *WEB* webcam-delivered telepsychiatry; MQRS scale rating 1–6 low quality, 7–13 moderate, 14–16 high quality

**Table 2:** Interventions/outcomes

Study (first author, publication year)	Comparison type	Intervention	Control	Outcomes	Results
Dennis (2020)	Telemental health + in-person vs in-person control ^(2)^	12 weekly, 60 min nurse-delivered telephone interpersonal psychotherapy (IPT) sessions + standard available care	Standard locally available in-person post-partum care, including postpartum depression services from public health nurses, physicians, and community resources	- Primary: clinical depression at 12 weeks - Secondary: comorbid anxiety, self-reported attachment, partner relationship quality	- SCID depression: less in IPT up to 24 weeks - EPDS score > 12: less in IPT up to 24 weeks - Comorbid anxiety and partner relationship quality favored in IPT - 0 IPT responders relapsed - Attrition rate was low at < 20%
Celano (2020)	Telemental health vs telehealth control ^(4)^	4-week, study-trainer telephone-delivered positive psychology (PP) post-discharge	Weekly, clinician telephone-delivered phone calls, recalling neutral events post-discharge	- Feasibility: number of exercises completed - Acceptability: five-point Likert-type scales of ease and utility - Psychological constructs at 4- and 8-weeks post-enrollment	- PP group completed an average 3/4 PP exercises - PP exercises helpful - PP group trended towards improved positive affect and optimism
Chong (2011)	Telemental health vs in-person ^(1)^	Monthly, webcam-delivered telepsychiatry sessions (WEB)	In-person, treatment as usual (TAU) from psychiatrists	- Acceptability: appointment keeping, working alliance with provider, visit satisfaction, and antidepressant use - Feasibility: depression outcomes, functional days	- Working alliance with the psychiatrist and visit satisfaction higher in WEB group - More WEB patients used antidepressants - Depression severity decreased faster among WEB group - No difference in overall depression score
D’Souza (2002)	Telemental health vs in-person control ^(3)^	Inpatients received team-based videoconference discharge planning, then received 6 sessions of psycho-educational program	Inpatients received conventional in-person discharge summaries and no psycho-educational program	Treatment adherence, readmission, medication side effects, patient satisfaction	- More patients in the control group were readmitted over 12-months and reported medication side-effects - Patients in telemedicine group reported greater treatment adherence and more satisfaction with treatment and discharge planning
Egede (2015)	Telemental health vs in-person ^(1)^	8 sessions of video-delivered behavioral activation for MDD	8 sessions of in-person behavioral activation for MDD	Treatment response according to the Geriatric Depression Scale (GDS), Beck Depression Inventory (BDI), and Structured Clinical Interview for DSM-IV	- All 3 treatments responses did not differ significantly between groups; intention-to-treat population results were similar - No significant differences existed between treatment trajectories over time for BDI and GDS
Egede (2016)	Telemental health vs in-person ^(1)^	Video-based telemedicine MDD treatment	In-person MDD treatment	Quality of life (QoL), satisfaction, treatment credibility, and service delivery perception scores	- No significant difference in QOL, patient satisfaction, or treatment credibility - Retention was excellent within the range of (65–85%)
Fann (2015)	Telemental health vs in-person control ^(3)^	Brief cognitive behavioral therapy administered over the telephone (CBT-T)	Usual care (UC), in-person	Depression severity using Hamilton Depression Rating Scale (HAMD-17) and the patient-reported Symptom Checklist-20 (SCL-20) over 16 weeks	- No difference between combined CBT and UC groups over 16 weeks on HAMD-17 - CBT intervention reported symptom improvement and greater satisfaction with depression care
Lam (2013)	Telemental health vs telehealth control ^(4)^	Telephone-administered cognitive-behavioral therapy (telephone CBT) + escitalopram 10–20 mg/day	Adherence-reminder telephone calls + escitalopram 10–20 mg/day	Montgomery–Åsberg Depression Rating Scale (MADRS) and self-rated work functioning scales	- No difference in MADRS score improvement or in response/remission rates - Telephone-CBT group had greater improvement on work functioning -TMH high attrition rate of 33%
Mohr (2011)	Telemental health vs in-person control ^(3)^	16 sessions of telephone-administered cognitive behavioral therapy (T-CBT) over 20 weeks	In-person treatment as usual through the community-based outpatient clinic (CBOCs)	Hamilton Depression Rating Scale, the Patient Health Questionnaire-9, and a standardized psychiatric interview	- No time × treatment effects - 92.7% completed at least 12 sessions - 78.0% had no missed sessions
Schulze (2019)	Telemental health + CAU vs in-person CAU control ^(2)^	Proactive, regular telephone calls every second week for 6 months from three specially trained nurses	Care as usual: basic medical care involving occasional in-person visits to a physician to evaluate convalescence	Medication adherence via the Medication Adherence Report Scale	- Intervention group was significantly more likely to be medication adherent at 6 months - Social desirability, diagnosis, and medication did not affect the results
Yeung (2016)	Telemental health vs in-person control ^(3)^	Telepsychiatry-based culturally sensitive collaborative treatment (T-CSCT) for 6 months, involving cultural consultation via videoconference and care management	In-person treatment as usual (TAU) for 6 months	- Primary: 17-item Hamilton Depression Rating Scale (HAM-D) - Secondary: Clinical Global Impressions-Severity of Illness (CGI-S), Improvement (CGI-I) scales, Quality of Life Enjoyment and Satisfaction Questionnaire (Q-LES-Q)	- Odds of achieving response and remission were greater for the T-CSCT group - T-CSCT group had greater improvement over time in HAM-D and CGI-I scores
Luxton (2016)	Telemental health vs in-person ^(1)^	8 sessions of behavioral activation treatment for depression (BATD) at home via teleconferencing (HBTBH)	8 sessions of BATD in-person	- Primary: hopelessness and depression scores - Secondary: PTSD severity, depression diagnosis, and anxiety severity	- Noninferiority of HBTBH compared to in-person treatment based on hopelessness and depression scores - No difference in MDD diagnosis - Attrition rate of 32.23%
Heckman (2017)	Telemental health vs in-person control ^(3)^	9-weekly, 1 h telephone IPT treatments (tele-IPT)	Standard in-person care	- Primary: 4- and 8-month follow-up assessing depressive symptoms - Secondary outcome: interpersonal problems and social support	- Tele-IPT patients had significantly fewer depressive symptoms and interpersonal problems at post-intervention
Dobkin (2020)	Telemental health + TAU vs in-person control ^(2)^	Telephone-based CBT (T-CBT) + TAU	Community-based, in-person treatment as usual (TAU)	- Primary: mood and depression - Secondary: anxiety, QoL, durability of treatment effects, moderators of treatment response	- T-CBT outperformed TAU on all depression, anxiety, and QoL measures - T-CBT had improvements in mood (HAM-D)
Kalapatapu (2014)	Telemental health vs in-person ^(3)^	Telephone-administered CBT (T-CBT)	In-person CBT	Depression improvement, antidepressant use, Alcohol Use Disorders Identification Test, Treatment adherence	- No difference in all treatment adherence or depression outcomes at all time points
Hilty (2007)	Telemental health vs telehealth control ^(4)^	Intensive disease management modules (IDMM) using telephone, questionnaires, and repeated tele-video psychiatric consultation	Usual care with a DMM using telephone and self-report questionnaires	Depressive symptoms, physical functioning, role functioning, overall mental health, perception of general health, and bodily pain	- Clinical improvement for depression in both groups, with a significant trend in the more intensive module - Satisfaction and retention higher in intensive group
Hulsbosch (2017)	Telemental health vs in-person control ^(1)^	Videoconferencing (VCF) in outpatient care for SMI, available 24/7	Care as usual, in-person	- Primary: patient satisfaction - Secondary: QoL, loneliness, daily functioning, and the fulfillment of needs of care	- VCF had a positive effect on patient satisfaction; secondary outcomes showed no difference between groups

Group 1: direct comparison of same treatment delivered via telemental health vs in-person

Group 2: add-on to usual care vs usual care

Group 3: comparing some specific telemental health treatment to in-person control (not direct comparison)—cannot conclude that differences relate to the delivery as opposed to the content

Group 4: comparing one telemental health treatment to another—now we are looking at comparison of the content with the tele delivery being constant

Abbreviations: *MDD* major depressive disorder, *SMI* serious mental illness, *TAU–T-CBT* cognitive behavioral therapy, *TCBT* telephone-administered cognitive behavioral therapy, *VCF* videoconferencing, *IDMM* intensive disease management modules, *TAU* treatment as usual, *BATD* behavioral activation treatment for depression, *Tele IPT* telephone-based interpersonal psychotherapy, *HBTBH* home-based telebehavioral health, *EPDS* Postnatal Depression Scale, *SCID* clinically depressed, *CSCT* culturally sensitive collaborative treatment, *UC* usual care, *HAMD-17* Hamilton Depression Rating Scale, *QoL* quality of life, *PP* positive psychology, *MADRS* Montgomery–Åsberg Depression Rating Scale

### Sample Characteristics

A total of 1985 participants with mood disorders were analyzed across the 17 papers. Sample age ranged from 43 to 65 years old and gender ranged from 2.5 to 100% of the participants being female. Two papers reported ages as quartiles and thus were not included in the age range.^16,20^ Two publications reported different outcomes on the same participants (*n* = 241).^13,14^ Baseline demographics such as age, gender, sample size, population, location, study setting, and study duration are reported in Table 1.

Fourteen of the 17 papers (82.4%) included MDD patients^11,12, 14–17, 19, 20, 22–27^ and four included BD patients (23.5%).^13,14, 18, 21^ Lastly, one article included both MDD and BD (5.9%) patients.^14^

Six of the papers specifically investigated TMH in rural locations (35.3%).^11,15, 20, 21, 25, 27^ Twelve papers were conducted in the USA (70.6%).^11–13, 15–17, 19, 22–25, 27^ Other represented countries included Canada (*n* = 2, 11.8%), Australia (*n* = 1, 5.9%), Germany (*n* = 1, 5.9%) and the Netherlands (*n* = 1, 5.9%).^21,14, 18, 20, 26^

Six of the studies were conducted in community health clinics (35.3%).^11,12, 14, 15, 22, 23^ Four were set in inpatient psychiatric units (23.5%).^13,18, 19, 21^ Three took place in primary care settings (17.6%).^24,17, 27^ The remainder of the studies took place at a military facility (*n* = 1, 5.9%), an AIDS service organization (*n* = 1, 5.9%), and a referral service (*n* = 1, 5.9%). The study duration ranged from 4 weeks to 18 months (mean = 27.9 weeks). All 17 selected papers obtained baseline data prior to implementation of the remote delivery care intervention.

### Outcomes

#### Mood Outcomes

All 17 papers measured at least one clinical outcome (clinical depression, treatment response, medication adherence, patient acceptability, etc.). The majority of the papers (*n* = 12) had a primary outcome of change in mood symptoms among the following groups: TMH compared to in-person care alone, and the same treatment delivered via TMH and in-person. In the same intervention delivered via telehealth vs in-person comparison group, Chong found that while depression scores decreased more rapidly among treatment delivered via a video webcam vs in-person treatment group, there was no statistically significant difference in the depression score between the two groups.^15^ Luxton reported relatively strong and similar reductions in depression symptoms, hopelessness, and PTSD symptoms across both traditional office and videoconferencing groups comparing the same intervention.^16^ Egede found no significant differences between in-person treatment CBT compared with tCBT trajectories over time on the self-reported Beck Depression Inventory (BDI) and Geriatric Depression Scales (GDS).^11,12^

Two papers focused on telephone-administered cognitive behavioral therapy (tCBT) compared to in-person CBT control.^22,23^ Fann et al. found significant improvement on the patient-reported symptom checklist and greater satisfaction with depression care compared to the in-person group *p* < 0.001; however, there were no differences in clinician-rated Hamilton Depression Rating Scale (HAM-D) scores between the groups.^22^ Mohr did not find any significant difference in depression outcomes as measured by the clinician-rated HAM-D between patients receiving in-person treatment versus patients receiving tCBT.^23^

Three papers observed TMH treatment added on to usual care versus in-person usual care. Dobkin reported significant self-reported improvements in mood with tCBT compared to treatment as usual (TAU) which was defined by psychiatric consultations, medication management, supportive psychotherapy, and all aspects of routine care.^19^ Dennis examined clinical depression outcomes in post-partum patients between TMH interpersonal psychotherapy (IPT) plus in-person treatment versus in-person treatment alone.^20^ In this article, Dennis found that patients treated with both IPT and standard care were 4.5 times less likely to be clinically depressed than individuals in the standard local postpartum care group.^20^

Three papers examined mood outcomes in TMH vs telehealth control groups. Celano found improved positive affect, as measured by the Positive and Negative Affect Schedule (PANAS), as well as improved optimism and significant reductions in hopelessness, as measured by a five-point Likert scale, in the telephone-delivered positive psychology group compared to patients receiving telephone calls recalling neutral events.^13^ Lam et al. did not find a significant difference in depression change scores based on the Montgomery–Åsberg Depression Rating Scale (MADRS) after 12 weeks between patients who received escitalopram + tCBT vs those who received escitalopram + control telephone-based medication reminders.^26^ Hilty found clinical improvement for depression in both groups that received either telephone-based intensive disease management modules (IDMM) and televideo-based psychiatric consultation versus telephone-based disease management (DMM) alone with a significant trend favoring the IDMM group.^27^

#### Medication Adherence

In three papers, medication adherence was a reported outcome. D’Souza reported increased treatment adherence, which resulted in fewer readmissions and relapses, when comparing inpatients who received team-based videoconference discharge planning and six telemedicine-based psycho-educational program sessions, compared to inpatients who received in-person discharge summaries and no psycho-educational program.^21^ In another study, Schulze found that patients who received regular telephone calls as stand-alone therapy from specially trained nurses were significantly more likely to be adherent to their medications at 6 months, according to the Medication Adherence Report Scale (MARS), compared to patients who only had intermittent in-person visits with a physician with no TMH intervention.^18^

Finally, Kalapatapu reported that in-person CBT care and tCBT did not significantly differ in the number of CBT sessions attended, treatment engagement, treatment completion, or discontinuation of treatment.^17^

#### Patient Satisfaction and Study Attrition

Patient satisfaction was a reported outcome in several included papers. Hulsbosch used the Geestelijke Gezondheidszorg (GGZ) Thermometer, a personalized patient satisfaction questionnaire, and found that videoconferencing compared to control in-person usual care had a positive effect on patient satisfaction.^14^ However, no significant difference was found between the two groups on quality of life, loneliness, daily functioning, or the fulfillment of needs of care.^14^ Similarly, Mohr reported that patients were significantly more satisfied with a 20-week program for depression using tCBT compared to treatment as usual.^23^ In addition, Lam stated that tCBT was well accepted with 79% of participants rating themselves as satisfied or highly satisfied with the therapy.^26^ In contrast, based on the Charleston Psychiatric Outpatient Satisfaction Scale (CPOSS), Egede et al. (2016) found no significant difference in patient satisfaction or treatment credibility between TMH MDD medication treatment vs in-person MDD medication treatment.^12^

Data attrition or retention was noted in seven papers which provided a proxy for acceptability for positive clinical outcomes in different comparison groups.^11,12, 16, 19, 20, 26, 27^ In the TMH vs same treatment in-person group, Egede reported good patient retention with a range between 65 and 85% and excellent session attendance.^11^ Luxton reported that a total of 42 out of 82 participants completed all eight sessions of the same treatment as in-person care, and 40 completed all eight sessions using TMH, with a total attrition rate of 32.3%, and indicating no significant difference in attrition between the two groups.^16^ In the TMH vs telehealth control group, Hilty reported that retention was superior in the intervention group and that older participants were more likely to complete the study.^27^ In contrast, Lam found that in-person therapy had significantly higher attrition compared to the tCBT group and concluded that TMH is an acceptable method for CBT when compared to a telehealth control.^26^

#### Study Quality Assessment

The MQRS results are found in Table 1. The scores ranged from 7 to 13 with higher scores reflecting greater the methodological quality. The average score was 10.29 ± 1.66. All 17 papers were defined as moderate study quality. Strengths in study quality included every study being an RCT and inclusion of pre-intervention baseline analysis. Limitations of the quality assessment included minimal multi-site locations (*n* = 3) and limited objective verification in the papers.

## Discussion

TMH is a critical tool in the delivery of mental health and has demonstrated its ability to increase access to care.^3^ TMH quickly expanded since the onset of the COVID-19 pandemic given travel restrictions and a greater need for mental health services by a limited pool of mental health clinicians.^28^ The transition to TMH happened out of necessity and did not necessarily incorporate the evidence base stemming from RCTs prior to the onset of the pandemic. This selective narrative review evaluated the efficacy of TMH for the treatment of individuals with serious mood disorders prior to the COVID-19 pandemic.

### Mood Outcomes

With respect to clinical outcomes, when comparing TMH interventions for mood disorders to in-person care of the same intervention, this review found that there was minimal to no difference in the magnitude of improvement of symptoms. Similar to a 2010 systematic review by García-Lizana and Ingrid Muñoz-Mayorga which concluded that videoconference-based treatment in depression yields similar results as face-to-face therapy,^29^ the results suggest that TMH was not inferior to in-person treatment modalities. When comparing TMH + in-person vs in-person alone for clinical depression in post-partum patients, the add-on group showed larger improvements in clinical depression compared to the control group, indicating that TMH when used as an add-on has the potential to amplify mood outcomes.^20^ In a parallel study conducted by Guaiana et al., a reduction of depressive symptoms was observed in both TMH and control groups in individuals with MDD.^30^ Guaiana also showed that although TMH leads to symptom improvement in TMH in patients with mood disorders similar to in-person care, factors of relationship building, such as empathy and alliance between patient and psychiatrist over video, need further investigation.^30^

### Medication Adherence

Among the papers in the current review that examined medication adherence as an outcome, adherence improved with TMH and there were fewer readmissions and relapses. This may have important implications among patients in remote and rural areas where access to professional care is an issue.^21^ Additionally, telemedicine interventions for adherence allow the provider to address nonadherence outside of clinic and between visits. A 2020 systematic review by Basit found similar results. Namely, that TMH interventions may improve medication adherence in patients with depression, bipolar disorder, and schizophrenia.^31^ As TMH becomes increasingly more prevalent in the post-COVID-19 era, TMH interventions can be used to enhance in-person treatment and aid in medication adherence across various psychiatric disorders. However, further research is needed to better understand the mechanisms of change and the specific content of effective TMH interventions.^31^

### Patient Satisfaction and Study Attrition

As for patient satisfaction, while not consistent, in two studies, satisfaction was greater in TMH groups than same treatment in-person groups.^14,15^ A 2020 systematic review found that patient satisfaction is equivalent or significantly higher in TMH than face-to-face intervention in patients with MDD.^30^ Benefits of TMH that improve patient satisfaction compared to in-person treatment include less travel time, minimizing time away from work, and improved scheduling convenience.^26^ Importantly, there is a need to determine which patient populations and particular patients are more likely to attend and follow through with TMH than with in-person care. In this way, there is the potential to engage more patients in the type of treatment that is most acceptable and suitable for them.

In several of the included studies, the feasibility of TMH use prior to the COVID-19 pandemic was measured through completed appointments and exercises, as well as service delivery perception.^12,13, 15^ TMH has been shown to reduce travel costs and increase attendance, particularly for patients living in areas where psychiatric services are not locally available.^23,27^ One prospective study revealed that most patients would be willing to pay for such a service at home.^4^ Potential barriers include user acceptance, older age, technological issues, and resistance among providers, all of which may inhibit the use of TMH.^15^

### Limitations and Strengths

While this review adds to the body of knowledge of TMH in mood disorders prior to COVID-19, there are several limitations that may impact generalizability. Less than 50% of the papers selected were carried out at multiple sites, all had generally small sample sizes, and there were a limited number of papers (*n* = 17). Furthermore, only 23.5% (*n* = 4) of the papers focused on individuals with BD, indicating that the conclusions may not be generalizable to this patient population and that further study of TMH for the treatment of BD is needed.^13,14, 18, 21^ Furthermore, the samples of participants are from predominantly Caucasian, wealthy, Western nations, and may not be generalizable to countries outside of those sampled in this paper. As most of these papers were conducted in highly industrialized countries, this may introduce bias as the population is likely to have better access to TMH compared to other countries. Finally, many of these studies utilized basic technology (e.g., telephone, early versions of webcams) which may not compare to post-COVID video platforms. While the manuscript provides an overview of telehealth’s potential in treating mood disorders, it falls short in concluding what works best for whom. There are several strengths of the narrative approach, however, that may in part offset methodological weaknesses. The inclusion of only RCTs helped to ensure a minimum of study rigor and better approximation of TMH outcome comparisons, while the global distribution of studies helped make conclusions more generalizable to the broader population of people with MDD and preliminary signal with BD.

### Future Areas of Research

The narrative review findings also suggest areas for future research. There were few studies that investigated comparisons of TMH versus other telehealth interventions or TMH plus in-person versus in-person interventions alone. These areas can be further explored to better understand the utility of TMH across various domains. Conducting well-designed RCTs comparing the use of higher technology TMH interventions in patients with serious mood disorders post-COVID-19 could significantly add to the scientific knowledge base. Other future studies might include a greater focus on patients with BD, with greater awareness of barriers to treatment adherence, TMH for patients with mood disorders in rural areas, and needed refinements in TMH delivery such as the determination of videoconferencing best practices and other TMH therapies.

## Implications for Behavioral Health

Use of TMH prior to the onset of the COVID-19 pandemic for treatment of serious mood disorders demonstrates the benefits of access to care and follow-up, with few apparent differences in clinical outcomes between TMH and in-person care. With the shift towards distance technology, TMH is a viable means for treating patients with MDD and possibly BD. Specific challenges for treating these patients may include barriers to technological access and how to augment TMH with needed in-person interventions such as medication injection or physical/neurological examination. These preliminary findings highlight the need for future work to investigate best use and practices for carrying out TMH for patients with serious mood disorders on a broader scale and in real-world settings.

## Supplementary Information

Below is the link to the electronic supplementary material.

Supplementary file1 (DOCX 15 KB)

## Abbreviations

BATD: Behavioral activation treatment for depression
IDMM: Intensive disease management modules
IPT: Interpersonal psychotherapy
MDD: Major depressive disorder
PP: Positive psychology
TAU: Treatment as usual
T-CBT: Telephone deliver cognitive behavioral therapy
TMH: Telemental health
WEB: Webcam-delivered telepsychiatry
VCF: Videoconferencing
