# Supplementary material for: The Efficacy of Telemental Health Interventions for Mood Disorders Pre-COVID-19: A Narrative Review
Source: J Behav Health Serv Res. 2024 May 2;51(3):395–420. doi: 10.1007/s11414-024-09884-5 (PMC11180629; doi:10.1007/s11414-024-09884-5)
Supplement: Supplementary file 1 — Supplementary file1 (DOCX 15 KB) [file 11414_2024_9884_MOESM1_ESM.docx]

**Appendix**

**PUBMED**

**Prelim (1/4/2021)**

(telemedicine[mesh] OR telemed*[tiab] OR telehealth*[tiab] OR telepsych*[tiab] OR teleconferenc*[tiab] OR teleconsult*[tiab] OR remote deliver*[tiab] OR remote consult*[tiab] OR virtual deliver*[tiab] OR virtual consult*[tiab]) AND ("serious mental illness*"[tiab] OR "severe mental illness*"[tiab] OR "depressive disorder, major"[mesh] OR major depression[tiab] OR major depressive[tiab] OR "bipolar and related disorders"[mesh] OR bipolar[tiab]) AND english[lang] 386

**1/15/2021**

(telemedicine[mesh] OR telemed*[tiab] OR telehealth*[tiab] OR telepsych*[tiab] OR teleconferenc*[tiab] OR teleconsult*[tiab] OR remote deliver*[tiab] OR remote consult*[tiab] OR virtual deliver*[tiab] OR virtual consult*[tiab]) AND ("serious mental illness*"[tiab] OR "severe mental illness*"[tiab] OR "depressive disorder, major"[mesh] OR major depression[tiab] OR major depressive[tiab] OR "bipolar and related disorders"[mesh] OR bipolar[tiab]) AND english[lang]

389

Published through Dec 31, 2019 → 330

Published beginning Jan 1, 2020 → 51

Overlap (epub/print) → 8

**COCHRANE**

**1/15/2021**

Search Name:

Date Run: 15/01/2021 15:38:45

Comment:

ID Search Hits

#1 MeSH descriptor: [Telemedicine] explode all trees 2618

#2 (telemed*):ti OR (telemed*):ab 1768

#3 (telehealth*):ti OR (telehealth*):ab 1168

#4 (telepsych*):ti OR (telepsych*):ab 114

#5 (teleconferenc*):ti OR (teleconferenc*):ab 251

#6 (teleconsult*):ti OR (teleconsult*):ab 135

#7 (remote deliver*):ti OR (remote deliver*):ab 809

#8 (remote consult*):ti OR (remote consult*):ab 277

#9 (virtual deliver*):ti OR (virtual deliver*):ab 574

#10 (virtual consult*):ti OR (virtual consult*):ab 165

#11 #1 OR #2 OR #3 OR #4 OR #5 OR #6 OR #7 OR #8 OR #9 OR #10 6396

#12 MeSH descriptor: [Depressive Disorder, Major] explode all trees 4977

#13 (major depression):ti OR (major depression):ab 12455

#14 (major depressive):ti OR (major depressive):ab 9772

#15 MeSH descriptor: [Bipolar and Related Disorders] explode all trees 2681

#16 (bipolar):ti OR (bipolar):ab 7705

#17 MeSH descriptor: [Schizophrenia] explode all trees 7572

#18 (schizophren*):ti OR (schizophren*):ab 16383

#19 (schizoaffective*):ti OR (schizoaffective*):ab 1769

#20 (serious mental illness*):ti OR (serious mental illness*):ab 1105

#21 (severe mental illness*):ti OR (severe mental illness*):ab 1608

#22 #12 OR #13 OR #14 OR #15 OR #16 OR #17 OR #18 OR #19 OR #20 OR #21 40847

#23 #11 AND #22 228

End 2019 → 193

Begin 2020 → 35

**WEB OF SCIENCE**

**1/15/2021**

# 4

237

#2 AND #1

Refined by: LANGUAGES: ( ENGLISH )

# 3

241

#2 AND #1

# 2

400,126

TS=("major depressive" OR "major depression" OR bipolar OR schizophren* OR schizoaffective* OR "serious mental illness*" OR "severe mental illness*")

# 1

32,241

TS=(telemed* OR telehealth* OR telepsych* OR teleconferenc* OR teleconsult* OR "remote deliver*" OR "remote consult*" OR "virtual deliver*" OR "virtual consult*")

End 2019 → 197

Begin 2020 → 40

**PSYCINFO**

**1/15/2021**

Database: APA PsycInfo <1806 to January Week 2 2021>

Search Strategy:

--------------------------------------------------------------------------------

1 exp telemedicine/ (9294)

2 telemed*.ti. or telemed*.ab. (1642)

3 telehealth*.ti. or telehealth*.ab. (1537)

4 telepsych*.ti. or telepsych*.ab. (676)

5 teleconferenc*.ti. or teleconferenc*.ab. (448)

6 teleconsult*.ti. or teleconsult*.ab. (178)

7 remote deliver*.ti. or remote deliver*.ab. (61)

8 remote consult*.ti. or remote consult*.ab. (26)

9 virtual deliver*.ti. or virtual deliver*.ab. (11)

10 virtual consult*.ti. or virtual consult*.ab. (20)

11 1 or 2 or 3 or 4 or 5 or 6 or 7 or 8 or 9 or 10 (10272)

12 exp major depression/ (135370)

13 major depression.ti. or major depression.ab. (22633)

14 major depressive.ti. or major depressive.ab. (25603)

15 exp bipolar disorder/ (31151)

16 bipolar.ti. or bipolar.ab. (39745)

17 exp schizophrenia/ (91945)

18 schizophren*.ti. or schizophren*.ab. (121292)

19 schizoaffective*.ti. or schizoaffective*.ab. (6514)

20 serious mental illness*.ti. or serious mental illness*.ab. (4739)

21 severe mental illness*.ti. or severe mental illness*.ab. (5303)

22 12 or 13 or 14 or 15 or 16 or 17 or 18 or 19 or 20 or 21 (297835)

23 11 and 22 (944)

24 limit 23 to english language (901)

***************************

End 2019 → 831

Begin 2020 → 70

**CINAHL**

**1/15/2021**

S24 S11 AND S23 Limiters - English Language 856

S23 S12 OR S13 OR S14 OR S15 OR S16 OR S17 OR S18 OR S19 OR S20 OR S21 OR

S22 166,761

S22 TI "serious mental illness*" OR AB "serious mental illness* 2,962

S21 TI "severe mental illness*" OR AB "severe mental illness*" 2,975

S20 TI schizoaffective* OR AB schizoaffective* 1,659

S19 (MH "Schizoaffective Disorder") 335

S18 TI schizophren* OR AB schizophren* 27,644

S17 (MH "Schizophrenia+") 25,975

S16 TI bipolar OR AB bipolar 15,262

S15 (MH "Bipolar Disorder+") 11,986

S14 TI "major depressive" OR AB "major depressive" 9,008

S13 TI "major depression" OR AB "major depression" 6,575

S12 (MH "Depression+") 115,374

S11 S1 OR S2 OR S3 OR S4 OR S5 OR S6 OR S7 OR S8 OR S9 OR S10 29,606

S10 TI "virtual consult*" OR AB "virtual consult*" 71

S9 TI "virtual deliver*" OR AB "virtual deliver*" 14

S8 TI "remote consult*" OR AB "remote consult*" 120

S7 TI "remote deliver*" OR AB "remote deliver*" 51

S6 TI teleconsult* OR AB teleconsult* 475

S5 TI teleconferenc* OR AB teleconferenc* 679

S4 TI telepsych* OR AB telepsych* 468

S3 TI telehealth* OR AB telehealth* 4,494

S2 TI telemed* OR AB telemed* 5,934

S1 (MH "Telehealth+") 26,363

End 2019 → 737

Begin 2020 → 119
